# Thermodynamic Balance vs. Computational Fluid Dynamics Approach for the Outlet Temperature Estimation of a Benchtop Spray Dryer

**DOI:** 10.3390/pharmaceutics14020296

**Published:** 2022-01-27

**Authors:** Andrea Milanesi, Francesco Rizzuto, Maurizio Rinaldi, Andrea Foglio Bonda, Lorena Segale, Lorella Giovannelli

**Affiliations:** 1 Department of Pharmaceutical Sciences, Università del Piemonte Orientale, 28100 Novara, Italy; andrea.milanesi@uniupo.it (A.M.); maurizio.rinaldi@uniupo.it (M.R.); andrea.fogliobonda@uniupo.it (A.F.B.); lorena.segale@uniupo.it (L.S.); 2 Department of Mechanical and Aerospace Engineering, University of Strathclyde, Glasgow G1 1XQ, UK; francesco.rizzuto@strath.ac.uk

**Keywords:** spray drying, design space, scale-up, modeling, process modeling, outlet temperature, a-dimensional parameter, machine learning

## Abstract

The use of design space (DS) is a key milestone in the quality by design (QbD) of pharmaceutical processes. It should be considered from early laboratory development to industrial production, in order to support scientists with making decisions at each step of the product’s development life. Presently, there are no available data or methodologies for developing models for the implementation of design space (DS) on laboratory-scale spray dryers. Therefore, in this work, a comparison between two different modeling approaches, thermodynamics and computational fluid dynamics (CFD), to a laboratory spray dryer model have been evaluated. The models computed the outlet temperature ($T_{\mathrm{out}}$) of the process with a new modeling strategy that includes machine learning to improve the model prediction. The model metrics calculated indicate how the thermodynamic model fits $T_{\mathrm{out}}$ data better than CFD; indeed, the error of the CFD model increases towards higher values of $T_{\mathrm{out}}$ and feed rate (FR), with a final mean absolute error of 10.43 K, compared to the 1.74 K error of the thermodynamic model. Successively, a DS of the studied spray dryer equipment has been implemented, showing how $T_{\mathrm{out}}$ is strongly affected by FR variation, which accounts for about 40 times more than the gas flow rate ($G_{\mathrm{in}}$) in the DS. The thermodynamic model, combined with the machine learning approach here proposed, could be used as a valid tool in the QbD development of spray-dried pharmaceutical products, starting from their early laboratory stages, replacing traditional trial-and-error methodologies, preventing process errors, and helping scientists with the following scale-up.

## 1. Introduction

Spray drying is a single-step powder-manufacturing process dominated by the evaporation of liquid systems, and it is widely applied in the pharmaceutical, biomedical, food, and cosmetic industries [1,2,3,4,5]. From its invention in 1872 by Samuel Percy, this technology has been further developed to produce powders with improved properties while maintaining relatively low production costs [1]. Over the past 15 years, spray drying has been successfully applied in the manufacturing of pharmaceutical proteins, and its use has been firmly established as a producer of amorphous solid dispersions, enhancing the bioavailability of poorly soluble drugs [6,7]. Moreover, it is useful for drugs for controlling taste-masking or producing dosage forms that can be administered by inhalation [7]. Although the spray-drying process is a simple technique for obtaining a powder, the droplet-drying kinetics and subsequent thermal events are complex. The liquid feed, consisting of solutions, emulsions, or suspensions, enters the spray dryer and undergoes a series of transformations. First, the liquid is sprayed by the atomizer, and then it is mixed with a hot airflow, resulting in the drying of the droplets [8]. This drying process occurs in the tower of the equipment. Each droplet is, first, heated to the wet-bulb temperature. Then, the drying process starts from the surface of the droplet, while at the same time, the solvent molecules migrate through the surface. During this first part of the drying process, adiabatic evaporation occurs, defined as the constant-rate drying stage [9]. A second step, the falling-rate period, begins when the solvent is mainly evaporated and the product concentration at the droplet’s surface increases with a crust formation. This event slows the drying rate of the droplets and leads to the formation of a solid particle, with the concomitant increase of the temperature until it reaches the surrounding gas temperature. Subsequently, the cooled and humidified gas moves together with the dried product towards a downstream cyclone, where they are then separated [10]. Due to the complexity of this process, the final dried product’s characteristics are determined by the drying rate and can be influenced by several parameters. Therefore, the key factors of the overall system are: the inlet temperature of the drying gas ($T_{\mathrm{in}}$), the feed flow rate (FR), the feed concentration ($c_{\mathrm{feed}}$), and the mass flow rate ($G_{\mathrm{in}}$). Although other parameters such as the solid content of the slurry or the tower dimensions are less controllable, they play an important role in the maximum achievable particle size or in the residence time of the particles. The outlet temperature of the drying gas ($T_{\mathrm{out}}$) is one of the most important points to consider in spray-drying processes [1,11] as its variation affects several other phenomena directly connected to the product quality. For example, maintaining constant FR, $G_{\mathrm{in}}$, and $c_{\mathrm{feed}}$, the $T_{\mathrm{out}}$ variation influences the residual moisture of the product [12]; this evidence is particularly important, as a high level of residual moisture increases the microbiological activity and promotes product stickiness [8]. To obtain a product with lower residual moisture, generally, $T_{\mathrm{in}}$ must be as high as possible; nevertheless, setting the temperature at the highest level is not always the right approach due to the processing of thermolabile bioproducts and the desire to preserve the active substance from thermal degradation [12]. Furthermore, the increase of the temperature and, thus, of the evaporation rate further reduces molecular mobility in the droplets, which leads to high Pèclet number values, resulting in an irregular shape with a deeply affected morphology of the particles [10]. The highest temperature the product will experience during the drying process will be $T_{\mathrm{out}}$, and this parameter has been reported as crucial in the prevention of thermal stress in spray-dried products such as proteins [6,13]. Another phenomenon directly connected to the thermal processes, and in particular to $T_{\mathrm{out}}$, is the glass-transition temperature ($T_{g}$) of the processed products, that is the temperature above which the particle structure turns from a glassy to a rubbery state. In general, the outlet air temperature should be 10 °C below the $T_{g}$ of the droplet’s surface layer to prevent product stickiness, depositions on the internal surfaces of the spray dryer, and quality degradation [14]. Taking into consideration that the aforementioned phenomena are deeply affected by $T_{\mathrm{out}}$ during a scale-up process, maintaining the same $T_{\mathrm{out}}$ between different equipment sizes is the most-used approach. Nevertheless, the transfer from laboratory- to production-scale presents big differences in the spray dryers’ characteristics. The evaporation rate capacity, gas flow rate capability, residence time, and thermal insulation do not scale proportionally with the size of the dryer, making the study more challenging to achieve the same results [7]. However, as this temperature is strongly dependent on other process parameters, many experiments are required, leading to a considerable increase in costs and wasted materials especially in pharmaceutical applications [15]. Therefore, the trial-and-error procedure to transfer $T_{\mathrm{out}}$ from one equipment size to another results in significant time consumption and the waste of manpower and raw materials. In the development of a pharmaceutical manufacturing process, it is important to adopt an accessible solid strategy capable of providing a complete identification of the critical process variables involved. A valid approach is the design space (DS); its quality is a key feature, in the pharmaceutical context, for describing the process parameters appropriately, allowing study of the parameters that deeply affect the product quality [16]. The importance of DS is highlighted also by the ICH Q8 guideline, which defines it as a “multidimensional combination and interaction of input variables (e.g., material attributes) and process parameters that have been demonstrated to provide assurance of quality” [17]. For industries, the DS is strategically noteworthy. It implies that as long as the process is kept within the defined DS limits, the process and the quality of the final product will be known, and no regulatory post-approval change will be required [18]. Using modeling tools to obtain a DS can lead to an effortless scale-up with a better prediction of the final product properties, as proposed in a paper by Lebrun et al., where a DS that takes into account multiple critical quality attributes of the product has been performed [18]. Another example of the DS approach has been used for the development of an amorphous solid dispersion, in order to avoid risk of API crystallization and enhance its stability [19]. In spray drying, DS composite variables, formed by single parameters, are usually considered to allow two-dimensional representations of the DS. The most useful composite variable used in the spray-drying process is $FR/G_{\mathrm{in}}$, which entails an a-dimensional parameter [20]. Furthermore, suitable combinations of multiple input variables can be constructed to obtain informative 2D representations, having as outputs $T_{\mathrm{out}}$ or the relative humidity (RH). To reduce the number of experiments and understand the key factors for obtaining specific powder characteristics, the use of mathematical models is embraced. Well-established models are present in the literature; Poozesh and collaborators give a comprehensive overview of the different modelling approaches for the spray-drying process [7]. A more complex and accurate description of the spray-drying phenomena can be conducted with a three-dimensional analysis of the fluid flow. This method requires solving the full set of fluid dynamic equations, which leads to an enormous amount of computational effort. However, this approach is valuable when a detailed description of the fluid pattern or the optimal design configuration is required [7]. Another possibility is the use of mass and energy balance approaches, which allow the evaluation of the mass and energy transfers during the process and can be used for predicting and setting a reproducible drying condition for the feed dehydration. These tools can also be used to set a DS of a specific spray dryer, and several authors proposed this approach for an industrial plant [20,21]. However, even if the thermodynamic balance was used on smaller spray-drying scales [22,23], no DS was performed.

Therefore, this work aims to fulfill the lack, in the literature, of data and methodologies for developing DS for small-scale spray dryers. The emphasis is given to a unique comparison between the thermodynamic and the computational fluid dynamic (CFD) approach, evaluating the $T_{\mathrm{out}}$. In addition to improving the thermodynamic approach, a machine learning technique was included to obtain consistent results; moreover, all data are experimentally validated.

## 2. Materials and Methods

### 2.1. Experiments and Measurements

A Büchi B-290 laboratory-scale spray dryer, manufactured by Büchi Labortechnik AG (Flawil, Switzerland), was used for the experiments. Additional instruments were used to include more data. A Pitot anemometer (TROTEC TA400, Trotec GmbH, Heinsberg, Germany) was placed upstream of the heater to map the effective drying gas speed in m/s ($G_{\mathrm{in}}^{m}$), and the gas flow rate capacity of the blower in kg/h ($G_{\mathrm{in}}$), with its variation at different air temperatures. A display pressure switch (PSD, Kobold Messring GmbH, Hofheim am Taunus, Germany) was placed between the nozzle and the spray dryer to obtain a precise evaluation of the atomization gas pressure. To avoid significant variation of the inlet air humidity, the entering inlet air was previously dehumidified (Dehumidifier B-296 Büchi Labortechnik AG, Flawil, Switzerland). The experiments were conducted when the spray dryer had reached the set point $T_{\mathrm{in}}$ and the $T_{\mathrm{out}}$ was stabilized with a variation of ±1 °C in 10 min. The same criteria were applied to record the $T_{\mathrm{out}}$ data during atomization. The calibration of the peristaltic pump used for feeding the liquid was checked daily with deionized water.

### 2.2. Experimental Design

Two sets of experiments were designed to define the spray dryer’s operating range. The first one consisted in evaluating the performances with a selection of empty runs (i.e., without the drying of any liquids). The experimental points were defined with 4 levels of $T_{\mathrm{in}}$ and 3 levels of aspirator rate (i.e., blower speed) respectively, 473, 433, 413, and 373 K, and 100%, 80%, and 50%. The second set of experiments was conducted to identify the limits of the operating range of the spray dryer in “atomizing condition” (i.e., drying deionized water where $c_{\mathrm{feed}}$ was zero). This evaluation of the evaporation rate efficiency was visually performed during the atomization process, with an inspection of the colliding droplets at the bottom of the tower. Only when the droplets visible on the bottom surface of the tower were negligible, the evaporation of the solvent was considered completed, and so the experiment’s process conditions were considered within the spray dryer’s operating range. Each experimental point was conducted in triplicate, keeping the atomization air pressure constant at 6 bar and monitoring the environmental conditions of relative humidity ($RH_{\mathrm{ext}}$) and temperature ($T_{\mathrm{ext}}$) with a thermohygrometer (TROTEC BC21, Trotec GmbH).

### 2.3. Thermodynamic Model Development

The experimental points were used to create a dataset for the thermodynamic model. The spray dryer was simplified as a cylinder, and the following assumptions were made:

- air is treated as an ideal gas;
- steady-state conditions;
- the inner surface of the spray dryer is smooth;
- the thermal conductivity values of the air ($cp_{\mathrm{gas}}$) at $T_{\mathrm{in}}$ is linear in the range of the experimental temperatures [24];
- the heat transfer coefficient of inside convection can be calculated using the average temperature $T_{\mathrm{avg}}=\left(T_{\mathrm{in}}+T_{\mathrm{out}}\right)/2$.

To compute $T_{\mathrm{out}}$ in the spray-drying process, an overall heat-balance approach was used, in which the control volume was the entire spray dryer between the inlet probe temperature and the outlet one. This approach has been developed from the energy balance (Equation (1)), which takes into consideration the energy input ($Q_{\mathrm{in}}$), the energy in the atomization and evaporation ($Q_{\mathrm{feed}}$), along with the energy output ($Q_{\mathrm{out}}$) and the loss of heat through the spray dryer’s walls ($Q_{\mathrm{loss}}$) [21], which is assumed to be the sum of lost energy by heat convection/conduction ($Q_{\mathrm{loss}}^{R}$) and radiation ($Q_{\mathrm{loss}}^{\mathrm{rad}}$):

(1) $$Q_{\mathrm{in}}=Q_{\mathrm{feed}}+Q_{\mathrm{loss}}+Q_{\mathrm{out}}\;$$

where:

(2) $$Q_{\mathrm{in}}=G_{\mathrm{in}}\cdot cp_{\mathrm{gas}}\cdot T_{\mathrm{in}}\;$$

(3) $$Q_{\mathrm{out}}=G_{\mathrm{in}}\cdot cp_{\mathrm{gas}}\cdot T_{\mathrm{out}}\;$$

dividing Equation (1) by:

(4) $$cp_{\mathrm{gas}}\cdot G_{\mathrm{in}}\;$$

Thus, Equation (5) has been obtained:

(5) $$T_{\mathrm{out}}=T_{\mathrm{in}}-\frac{Q_{\mathrm{feed}}+Q_{\mathrm{loss}}\left(T_{\mathrm{out}},T_{\mathrm{ext}}^{\mathrm{wall}},\ldots \right)}{cp_{\mathrm{gas}}\cdot G_{\mathrm{in}}}$$

where the dependencies from the variables $T_{\mathrm{out}}$ and the temperature on the spray dryer’s outer surface ($T_{\mathrm{ext}}^{\mathrm{wall}}$) have been made explicit. In Equation (5), the variable $T_{\mathrm{out}}$ appears not only on the left side but also through $Q_{\mathrm{loss}}$ on the right side of the equation. The knowledge of $T_{\mathrm{ext}}^{\mathrm{wall}}$ is necessary to use Equation (5), and it has been calculated separately using Equation (6):

(6) $$T_{\mathrm{ext}}^{\mathrm{wall}}(T_{\mathrm{in}},T_{\mathrm{out}},c_{\mathrm{feed}},T_{\mathrm{ext}},{RH}_{\mathrm{ext}},FR,G_{\mathrm{in}}^{m})={\left(\frac{Q_{\mathrm{loss}}^{\mathrm{rad}}\left(T_{\mathrm{in}},T_{\mathrm{out}},c_{\mathrm{feed}},T_{\mathrm{ext}},RH_{\mathrm{ext}},FR,G_{\mathrm{in}}^{m}\right)}{{\mathrm{Area}}_{\mathrm{ext}}\cdot \sigma \cdot {\mathrm{emissivity}}_{\mathrm{glass}}}+T_{\mathrm{ext}}^{4}\right)}^{1/4}$$

where σ is the Stefan–Boltzmann constant and ${\mathrm{Area}}_{\mathrm{ext}}$ is the external surface of the simplified cylinder.

**Figure 1:**
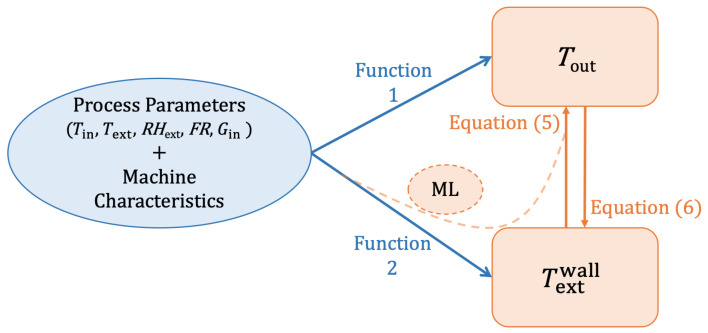
Schematic representation of the resolution approach used and the relationship between variables. The set of process parameters and spray dryer characteristics are shown in the blue oval, while the two experimental outputs linked by Equations (5) and (6) are expressed in the orange boxes. The machine learning (orange dashed line) allows the mimicking of Function 2 to determine $T_{\mathrm{ext}}^{\mathrm{wall}}$, and finally, to find, with Equation (5), $T_{\mathrm{out}}$.

For a better understanding, the algorithm strategy is reported in Figure 1, where the two experimental outputs, $T_{\mathrm{out}}$ and $T_{\mathrm{ext}}^{\mathrm{wall}}$, are related by Equation (5), in which $T_{\mathrm{out}}$ appears implicitly. The combination of experimental parameters and the spray dryer characteristics determines the $T_{\mathrm{out}}$ and $T_{\mathrm{ext}}^{\mathrm{wall}}$ values for each experiment. In principle, two different functions (Function 1 and 2) could be used to calculate $T_{\mathrm{out}}$ and $T_{\mathrm{ext}}^{\mathrm{wall}}$. If Function 2 is known, it could be possible to use Equation (5) to calculate $T_{\mathrm{out}}$; however, as the experimental outputs are available and include $T_{\mathrm{out}}$ but not $T_{\mathrm{ext}}^{\mathrm{wall}}$, it is possible to deduce $T_{\mathrm{ext}}^{\mathrm{wall}}$ using experimental $T_{\mathrm{out}}$ values by solving for $T_{\mathrm{ext}}^{\mathrm{wall}}$ Equation (5) (Equation (6)). Therefore, it is possible to construct an experimental dataset as the input along with their $T_{\mathrm{ext}}^{\mathrm{wall}}$ values as the output. This dataset has been used to mimic Function 2 through a machine learning (ML) approach. Then, since it is now possible to find the values of $T_{\mathrm{ext}}^{\mathrm{wall}}$ as a function of the experimental parameters, the solution path (dashed orange line in Figure 1) can allow the determination of $T_{\mathrm{out}}$.

Function 2 has been traced with a random forest algorithm starting with 5 features ($T_{\mathrm{in}}$, $T_{\mathrm{ext}}$, $RH_{\mathrm{ext}}$, FR, $G_{\mathrm{in}}$). All the experimental results were randomly divided into training and testing subsets. The training dataset contained 80% of the experiments, while the test dataset contained the remaining 20%. The performance of the model on the test dataset was evaluated by root mean square error (RMSE), R${}^{2}$, and mean absolute error (MAE). RMSE was used to select the optimal model. To enhance the accuracy of the ML prediction, additional experimental points were performed, with a total dataset of 74 points. The ML function, constructed with the experimental data, mimics Function 2 (Figure 1) and allows for the determination of $T_{\mathrm{ext}}^{\mathrm{wall}}$.

The substitution of $T_{\mathrm{ext}}^{\mathrm{wall}}$ in Equation (5) gives an implicit definition of $T_{\mathrm{out}}$. The solution of this equation has been achieved with an iterative numerical method, allowing it to mimic Function 1 (Figure 1). A maximum of 10 iterations have been performed on the entire function *h* (Equation (7)), where *h* is a shorthand for Equation (5), using $T_{\mathrm{in}}$ as starting value of $T_{\mathrm{out}}$:

(7) $$T_{\mathrm{out}}=h\left(T_{\mathrm{out}},T_{\mathrm{in}},\ldots \right).$$

### 2.4. CFD

**Figure 2:**
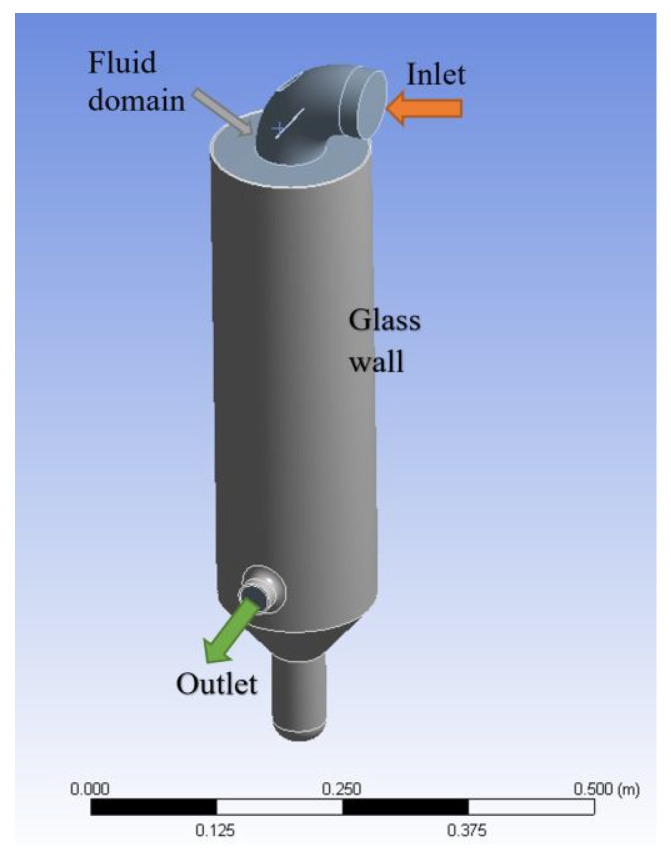
Three-dimensional representation of the simulated domain: the hot air is introduced from the inlet fluid boundary (orange arrow), and ejected with the water as a mixture from the outlet (green arrow). The glass wall boundary and the fluid domain are reported in grey and in light grey, respectively.

The fully three-dimensional fluid dynamics analysis was performed only to simulate the Büchi apparatus in the spray-drying process. First and foremost, a three dimensional CAD model was created and modified to obtain a simplified domain: holes, wall thickness thermal resistance, and seals were removed. Ansys software 18.2 was used to mesh and solve the system. The fluid-averaged model was computed with the Navier–Stokes set of equations. For the three-phase model (hot air, cold air, and water), the volume fraction model was applied and the droplet size was not taken into account directly. At the nozzle, the mass flow rate of the cold air and the water was averaged in terms of velocity, knowing the mass flow rate and the pressure of each phase. The entire domain was subdivided into 3.8 million cells and the analysis was performed by volume fraction methods. Three different fluids were considered at the same time: hot air, water, and vapour (when created). The evaporation phenomenon was evaluated by the Lee equation [25]. The viscous RANS model, in combination with the scalable wall function and thermal equation solved with the first-order upwind method, was used. The scalable wall function method includes a logarithmic treatment of the boundary layer to improve solution accuracy, rather than a linear function. To obtain the energy losses across the glass tower, two distinct domains were considered. The entry pipe and the inlet of the tower were assumed fully insulated and adiabatic. The tower was modelled with heat transfer across the glass walls with a wall thickness of 4 mm. The physical glass was also included in the simulation to improve the heat transfer exchange with the external ambient temperature (Figure 2). CFD simulations are not new in the spray drying field; Dobry and collaborators simulated the droplet and the dry particle movement in a much more simple environment [20]. However, differently from the previous research, the complexity of the model in this research took a step forward. The three-dimensional effects and the fluid-flow interaction were simulated. The simulations were considered concluded when the relative error of each equation reached $1\times 10^{-4}$, except for the volume fraction equations, where $1\times 10^{-3}$ was accepted. The following boundary conditions were imposed on the domain:

- at the inlet: the hot air velocity and temperature;
- at the nozzle: the uniform mixture (water and cold air) velocity, the temperature, and the volume fraction of the water;
- at the outlet: the ambient pressure.

Different from Cher Pin and Tee (2014), the simulation was conducted without 2D axial symmetry, and the model accounts for the temperature variation along the wall due to the flow path [26]. The asymmetry, due to the relative positions of the inlet and outlet duct, prevented the making of any geometric simplification.

### 2.5. Models Comparison

The calculated $T_{\mathrm{out}}$ data obtained from the models were compared with the experimental ones by RMSE, R${}^{2}$, MAE, and a residuals plot. The comparison was done on empty runs in atomizing conditions. The best model was used to plot the DS of the spray dryer by assigning $T_{\mathrm{out}}$ throughout the entire volume.

## 3. Results and Discussion

### 3.1. Experimental Results

Buchi B-290, thanks to its small dimension, is suitable as a laboratory spray dryer. However, it has numerous limitations, such as a lower drying capability, a short droplet/particle residence time in the drying tower that limits the achievable particle size under 20 microns, and the absence of a fine setting [27]. Despite these downsides, it is representative of dryers used in the R&D fields, and for this reason, it is a suitable starting point to develop the basis of our modelling approach. To realize the model based on the general Equation (1), it is necessary to estimate the heat lost by convection/conduction and radiation ($Q_{\mathrm{loss}}$) for this laboratory spray dryer. The $Q_{\mathrm{loss}}$ determination can be avoided by assuming that the spray dryer operates adiabatically and without heat losses. This assumption is feasible in the case of industrial sites where the equipment is insulated and, thus, presents a low overall heat transfer coefficient [19]. In laboratory equipment, to help the development and to allow for internal flow visualization, the tower is made of glass with an emissivity value between 0.62 and 0.95, implying a high $Q_{\mathrm{loss}}$; therefore, a radiation analysis should be performed [28]. Despite this consideration, the $Q_{\mathrm{loss}}$ estimation in this laboratory-scale equipment has been reported only for the heat loss due to convection [23]. Since $Q_{\mathrm{loss}}$ has the same importance as $Q_{\mathrm{feed}}$, it is mandatory to consider both these terms for an accurate $T_{\mathrm{out}}$ prediction. To estimate $Q_{\mathrm{loss}}$, the spray dryer was used without feed atomization; thus, the difference between the $T_{\mathrm{in}}$ and $T_{\mathrm{out}}$ was caused only by the heat loss of the equipment to the external environment. These empty-run experimental points are shown in Table 1, where the drying effect on the $T_{\mathrm{out}}$ can be observed; the higher the aspirator rate, the higher the $T_{\mathrm{out}}$ value at constant $T_{\mathrm{in}}$ that is observed. In addition, at high temperatures, a reduction in the gas flow was noticeable via the Pitot tube measurements. This relation between the mass of drying air and the blower aspiration rate has a non-linear behaviour at different temperatures. Indeed, at 473 K, the difference between the maximum and minimum suction speed is 10.1 kg/h, while it is 14.3 kg/h at 373 K. Despite the greater amount of drying gas circulating in the spray dryer at 373 K, the greatest difference between the $T_{\mathrm{out}}$ at the maximum and minimum blower aspirator rate was observed at the maximum level of $T_{\mathrm{in}}$ (Δ=9.3 K). This highlights the greater impact of the $G_{\mathrm{in}}$ parameter when the $T_{\mathrm{in}}$ is high, since small changes in $G_{\mathrm{in}}$ have a greater influence on $T_{\mathrm{out}}$.

The second set of experiments involving atomizing water (Table 2), was performed to find the limits of the spray dryer’s processing capability. Three process variables were considered: $T_{\mathrm{in}}$ in the range 373–463 K, $G_{\mathrm{in}}$ at its maximum and minimum, and FR starting from 0.07 kg/h. The limits for each temperature setting were found: at the highest temperature, the evaporation capability varies between 0.80 kg/h at the maximum aspiration rate and 0.42 kg/h at the minimum aspiration rate, resulting in the maximum a-dimensional ratio, AD = FR/$G_{\mathrm{in}}$, achievable from the spray dryer, $3.29\times 10^{-2}$. Conversely, the minimum AD of $2.29\times 10^{-3}$ was registered at 373 K, with the minimum FR (0.07 kg/h) and the maximum feasible $G_{\mathrm{in}}$. At the lower temperatures, 373, 403, and 433 K, the maximum evaporation capability at the maximum $G_{\mathrm{in}}$ was 0.23, 0.34, and 0.59 kg/h, while at the minimum it was 0.17, 0.21, 0.32 kg/h, respectively. These aforementioned limits in FR processing capability, as a function of different $T_{\mathrm{in}}$ and $G_{\mathrm{in}}$, along with the minimum value set of FR, describe a region of the spray dryer’s processing capability. The other five points, computed as the mean value of FR, $T_{\mathrm{in}}$, and AD of the limits defined above, were performed to verify the absence of colliding droplets at the bottom of the tower and, thus, the feasibility of the experimental points.

### 3.2. Thermodynamic Model

Using the described resolution approach to find $T_{\mathrm{out}}$, it is noticeable how the computation of $Q_{\mathrm{feed}}$, which can be represented as the heat spent in heating the product droplets to the wet-bulb temperature and the heat spent vaporizing the solvent, is straightforward and necessitates only the input parameters (Equation (S1)), see supplementary documentation). On the other hand, $Q_{\mathrm{loss}}$ estimation is more challenging: for its computation, and, thus, to solve Equation (5), it is necessary to know $T_{\mathrm{out}}$ and $T_{\mathrm{ext}}^{\mathrm{wall}}$ (Equation (S2)), see supplementary documentation). The former is the unknown term that represents the solution of Equation (5), while to measure $T_{\mathrm{ext}}^{\mathrm{wall}}$, suitable temperature sensors, which are hardly present as probe equipment in the laboratory-scale spray dryer, are necessary. For this reason, the resolution path, starting from the experimental inputs, is fundamental for determining the $Q_{\mathrm{loss}}$ term, which is composed of the sum of lost energy by convection/conduction ($Q_{\mathrm{loss}}^{R}$) and radiation ($Q_{\mathrm{loss}}^{\mathrm{rad}}$). To estimate the $Q_{\mathrm{loss}}^{R}$, the individual thermal resistances of the spray dryer wall against heat conduction and internal forced convection were treated as in series resistances [28]. The external natural convection term over the spray dryer surface was not considered for the $Q_{\mathrm{loss}}^{R}$ calculation. Its contribution was about 0.5% and, thus, was considered negligible in the $Q_{\mathrm{loss}}$ determination, where the major impact was imputable to the radiation energy loss. To calculate the emitted radiation, $Q_{\mathrm{loss}}^{\mathrm{rad}}$, from the spray dryer wall, the Stefan–Boltzmann law was applied [28]. The resulting $T_{\mathrm{ext}}^{\mathrm{wall}}$ was used to supervise the ML. The obtained $T_{\mathrm{ext}}^{\mathrm{wall}}$ values give a RMSE of 10.90 K, compared to the extrapolated ones ($R^{2}=0.69$, MAE = 8.49 K), leading to a satisfactory description of the experimental wall temperature in a laboratory spray dryer. The advantage of the ML using a random forest approach was to mimic the average surface temperature of the spray dryer wall at different conditions with more accuracy than other, simpler models. With the $T_{\mathrm{ext}}^{\mathrm{wall}}$ values, $T_{\mathrm{out}}$ was easily identified by using Equation (5) and applying a numerical iterative method, which converges quickly to the solution.

### 3.3. CFD

**Figure 3:**
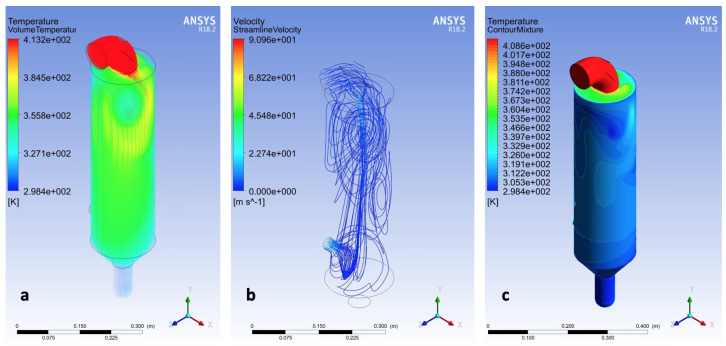
Example of simulation result for an inlet temperature of 413 K and water feed ratio of 0.426 kg/h. (**a**) Temperature distribution in the entire fluid volume, (**b**) velocity of the streamline starting from the inlet boundary, and (**c**) wall temperature.

CFD solutions were conducted for all key experimental points. An example of the results is reported in Figure 3. The temperature in the entire fluid domain and at the wall are plotted, respectively, in Figure 3a,b; meanwhile, the streamline velocity from the inlet boundary is depicted in Figure 3a. The temperature shows two distinct behaviours due to the subdomain division. The adiabatic zone is modelled at the inlet bent pipe and the tower zone, where the temperature depends on the fluid stream and the glass wall description.

The spatial temperature distribution on the tower surface is strongly dependent on the vortex flux in the tower. Although out of the scope of this research, this phenomenon points out the importance of the fluxes inside the spray dryer for optimization purposes. For this reason, the inlet pipe was not neglected from this simulation. No axial symmetry could be used to simplify the model; a major limitation in Pin et al. [26] is the description of the spray dryer in the three-dimensional analysis, which is complex and requires significant computational time. On the contrary, the main advantage is the clear description of the temperature, the velocity and, in the complex model, the moisture content in all the domains.

### 3.4. Models Comparison

**Figure 4:**
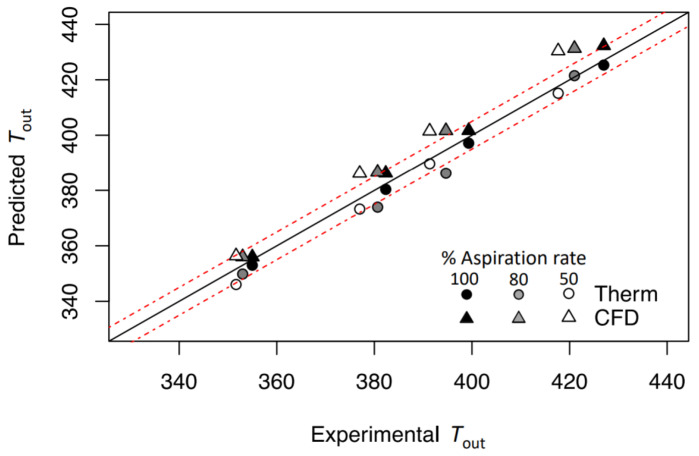
Comparison between predicted and observed values for the empty data set. Error bands are dashed in red at ±5 K from the reference value (solid black line).

**Figure 5:**
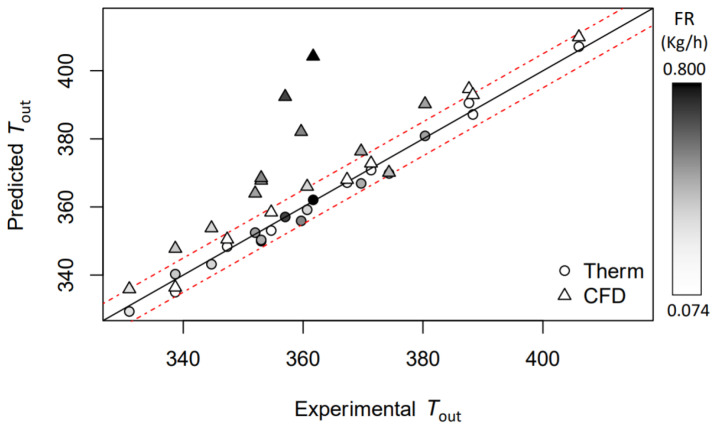
Comparison between predicted and observed values for the atomizing data set. Error bands dashed in red at ±5 K from the reference value (solid black line).

In this section, an in-depth discussion of the models is performed. Table 3, with Figure 4 and Figure 5, show the comparison between the complete CFD analysis and the thermodynamic model. For both models, the correlation with the experimental data is satisfying and presents an R${}^{2}$ of 0.99. Considering the RMSE and MAE, the CFD model is less accurate, with a higher discrepancy between the predicted and observed values. Looking at Figure 4, where the solid black line is the experimental reference and the dashed line represents the experimental values line ±5 K, it can be noticed that the CFD simulations are more consistent in terms of error. In detail, while there is an increase of $T_{\mathrm{out}}$, the CFD data is spread along *X* axis, in contrast to the thermodynamic model, where the data fit better around the solid line. The authors believe that the error in the CFD model increases while the aspiration rate decreases, mainly due to the model itself and its intrinsic turbulence description. It is important to point out that the thermodynamic model was ML-optimized to fit the experimental data. Differently, the CFD model did not undertake optimal data fitting or optimal heat-exchange studies. The response of the CFD model in atomizing conditions is different. The introduction of further equations (the Lee and vapor-fraction equations) increases the numerical complexity and convergence. Figure 5 shows the CFD model error increasing with the temperature and the injected water. When the water injection increases, the CFD model increases the difference from the experimental data. This provides a limitation on the CFD model for a higher fluid injection that must be further investigated. Moreover, a comparison is possible between the thermodynamic model and the results obtained by Lisboa et al., where the authors found a MAE of 0.80 K [21]. This value is 0.94 K lower than that obtained in the hereby presented model. However, unlike Lisboa et al., this result has been obtained with non-insulated equipment. This different condition generates higher thermal losses and, thus, greater uncertainty in the $T_{\mathrm{out}}$ determination within the model.

### 3.5. Spray Drying Design Space

**Figure 6:**
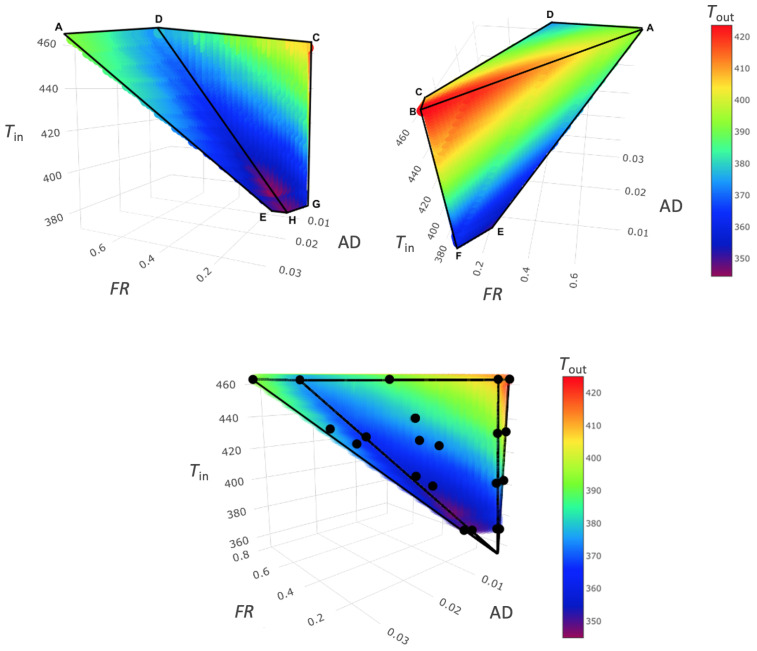
Design space volume and iso-level curve of $T_{\mathrm{out}}$, created with the thermodynamic model.

**Figure 7:**
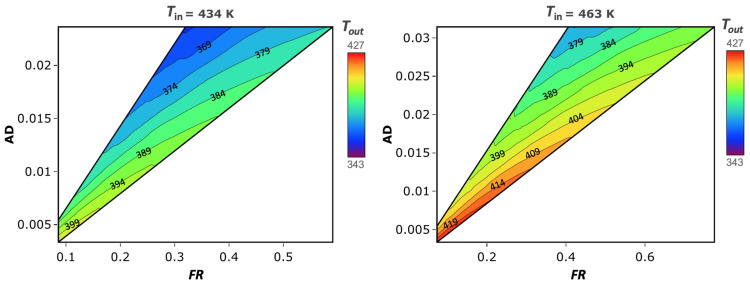
Contour plots of different DS volume sections at constant $T_{\mathrm{in}}$.

**Figure 8:**
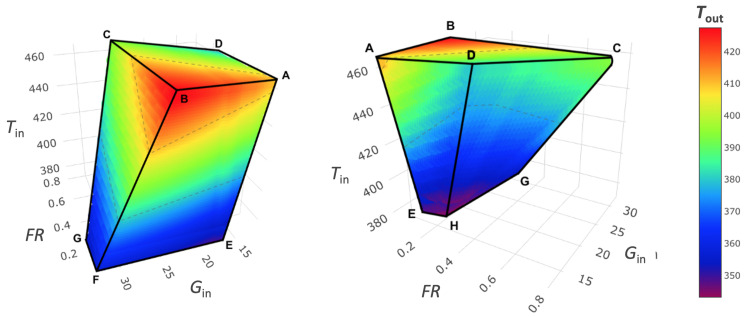
Design space volume and iso-level curve of $T_{\mathrm{out}}$, created with explicitated $G_{\mathrm{in}}$.

**Figure 9:**
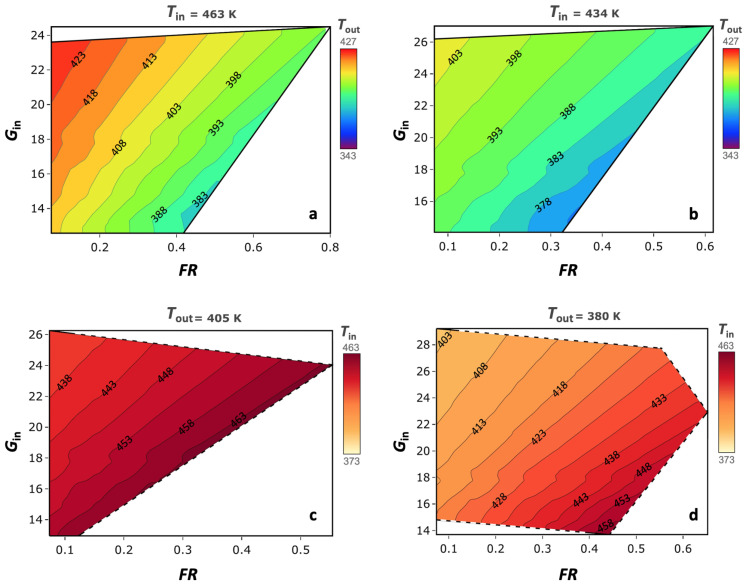
Contour plots of DS volume sections at two different $T_{\mathrm{in}}$, 463 and 434 K (**a**,**b**), and two $T_{\mathrm{out}}$, 405 and 380 K (**c**,**d**).

As shown by the second set of experiments, performed with atomizing water, it is possible to describe a region of the spray dryer’s processing capability. For each point of this region, a unique $T_{\mathrm{out}}$ can be found as a function of the experimental parameters ($T_{\mathrm{in}}$, $T_{\mathrm{ext}}$, $RH_{\mathrm{ext}}$, FR, and $G_{\mathrm{in}}$). This dependency can be reduced to three variables, considering that $T_{\mathrm{ext}}$ and $RH_{\mathrm{ext}}$ are fixed external environmental conditions temperature and relative humidity, respectively. Thus, the combination and interaction of these process parameters within this region origins a design space of the spray dryer $T_{\mathrm{out}}$, based on $T_{\mathrm{in}}$, FR, and AD. The developed thermodynamic model has shown a better accuracy compared to the CFD; hence, it has been used to create the design of space for the spray dryer. To ensure high resolution, a mesh of 22,241 points was developed. The resulting 3D working space is shown in Figure 6, where black points represent the aforementioned atomizing experiments while a rainbow scale is used to represent the variation of $T_{\mathrm{out}}$. The obtained volume is very close to a pyramid shape, with its vertex at a $T_{\mathrm{in}}$ of 354 K, outside the experimental ranges used. The reduction of the volume section at decreasing $T_{\mathrm{in}}$ in the graph represents a reduction in the FR processability and evaporation capability of the dryer at different temperatures. As clearly shown in the sections at a constant $T_{\mathrm{in}}$ (Figure 7), the AD parameter decreases when FR is fixed; thus, $G_{\mathrm{in}}$ is the maximum along the A–B side; on the other side, along C–D $G_{\mathrm{in}}$, is the minimum (Figure 6). The iso-level curves in the contour plots in Figure 7 show that the dependency of $T_{\mathrm{out}}$ is higher for FR than AD. The trends of $T_{\mathrm{out}}$ as a function of $T_{\mathrm{in}}$ and AD are qualitatively similar to those shown by Dobry et al. [20]. However, the AD operating range of this spray dryer is up to 10-times lower, due to smaller scale of the lab-scale spray dryer used. To better describe the impact of FR and $G_{\mathrm{in}}$, another representation (Figure 8) referred to $G_{\mathrm{in}}$, where $G_{\mathrm{in}}$ is reintroduced in place of AD, is given. From this volume, it is noticeable how rapidly $T_{\mathrm{out}}$ increases when FR decreases, and $G_{\mathrm{in}}$ increases at a constant $T_{\mathrm{in}}$. The selection of a single $T_{\mathrm{in}}$ from the volume allows us to better distinguish how the $T_{\mathrm{out}}$ changes on a face of the DS (Figure 9a,b). The volume sections show that the maximum growth trend of $T_{\mathrm{out}}$ is perpendicular to the iso-level curves of $T_{\mathrm{in}}$. This means that the minimum $T_{\mathrm{out}}$ is obtained by combining the maximum feasible FR with the minimum $G_{\mathrm{in}}$. The slope of the iso-level curves shows that decreasing FR by 0.1 kg/h is required to increase $G_{\mathrm{in}}$ by about 4 kg/h to maintain the same $T_{\mathrm{out}}$. The DS sections with constant $T_{\mathrm{in}}$ show similar iso-level curve slope values. The 2D representation obtained from the FR/$G_{\mathrm{in}}$ section of the plane at constant $T_{\mathrm{out}}$ is given in Figure 9c,d. For instance, two surfaces at $T_{\mathrm{out}}$ 405 K and 380 K are shown. From these plots, it is highlighted that the iso-level curves trend of $T_{\mathrm{in}}$ is different from the aforementioned sections shown in Figure 9a,b. Indeed, in the region of low $G_{\mathrm{in}}$, $T_{\mathrm{out}}$ is more sensitive to variation of $T_{\mathrm{in}}$ and FR, instead of variations of $G_{\mathrm{in}}$. Moreover, this phenomenon is non-linear, and to describe it, a higher-grade polynomial model should be used. In the region at low FR and $G_{\mathrm{in}}$, a degree of noise on the iso-level curves (Figure 9c,d) is depicted. Although further data may be included, the authors argue that this issue can be justified by the experimental data noise, which has a non-significant impact on the overall iso curves’ trend.

## 4. Conclusions

In this research, a complete CFD model approach was compared with a developed thermodynamic model and was experimentally validated. Although the CFD model is a good tool for optimizing and developing machine processes, as it allows for the study of the gas flow pattern that is particularly useful during scale-ups to small- or large-scale production, it shows limitations for the lab-scale spray dryer. Indeed, the thermodynamic approach developed allowed for a more precise description of $T_{\mathrm{out}}$. The thermodynamic solving path took advantage of machine learning to overcome some limitations of lab-scale spray dryer. This strategy can be easily applied to different equipment, as only a few steps are required, and data acquisition is normally performed during pre-heating and conditioning phases. The ability to predict the $T_{\mathrm{out}}$ was proven in this research. It represents a useful tool in the development and transfer of the spray-drying process across different scales. Although this model is at its beginning, further development and application on an insulated pilot-scale spray drier should be performed to understand the deepest impact of heat losses on the $T_{\mathrm{out}}$ estimation. In future works, the focus will be on the further data analysis of the design space, for instance, with additional information as the residual moisture dependency in the final product.

## Figures and Tables

**Table 1:** Spray dryer results of the empty runs, performed without feed atomization.

$T_{\mathrm{in}}$ (K)	% Asp	% ${\mathrm{RH}}_{\mathrm{ext}}$	$T_{\mathrm{ext}}$ (K)	$G_{\mathrm{in}}$ (kg/h)	$T_{\mathrm{out}}$ (K)
	100	25.6	294.0	23.8	427.0
473	80	34.4	299.1	20.4	421.0
	50	24.7	295.7	13.7	417.7
	100	29.5	293.7	27.8	399.3
433	80	22.3	297.5	22.5	394.7
	50	26.1	293.0	16.3	391.3
	100	43.7	297.4	28.4	382.3
413	80	35.7	298.0	24.5	380.7
	50	35.5	298.0	16.1	377.0
	100	32.6	294.2	33.8	355.0
373	80	24.8	298.5	26.9	353.0
	50	32.6	294.4	19.5	351.7

**Table 2:** Spray dryer’s processing limits obtained from atomizing water experiments.

$T_{\mathrm{in}}$ (K)	$T_{\mathrm{ext}}$ (K)	$T_{\mathrm{out}}$ (K)	% ${\mathrm{RH}}_{\mathrm{ext}}$ (K)	*FR* (kg/h)	$G_{\mathrm{in}}$ (kg/h)	*AD*
463	296	362	35.8	0.80	18.2	$3.27\times 10^{-2}$
	295	360	22.9	0.42	9.4	$3.29\times 10^{-2}$
	297	406	24.0	0.07	17.5	$3.12\times 10^{-3}$
	297	388	24.0	0.07	9.3	$5.86\times 10^{-3}$
433	297	357	40.9	0.59	18.2	$2.21\times 10^{-2}$
	295	353	30.3	0.32	9.4	$2.33\times 10^{-2}$
	298	388	23.7	0.07	18.7	$2.81\times 10^{-3}$
	298	371	22.8	0.07	9.8	$5.42\times 10^{-3}$
403	294	352	44.9	0.34	18.7	$1.18\times 10^{-2}$
	297	345	35.6	0.21	9.8	$1.37\times 10^{-2}$
	297	367	23.8	0.07	18.7	$2.55\times 10^{-3}$
	297	355	23.8	0.07	9.8	$4.85\times 10^{-3}$
373	295	339	41.7	0.23	19.2	$7.05\times 10^{-3}$
	295	331	29.1	0.17	10.3	$9.84\times 10^{-3}$
	297	347	24.2	0.07	19.3	$2.29\times 10^{-3}$
	297	339	24.2	0.07	10.1	$4.35\times 10^{-3}$

**Table 3:** Model evaluation metrics on $T_{\mathrm{out}}$ (K).

	RMSE (K)	$R^{2}$	MAE (K)
Empty Therm. model	4.04	0.99	3.34
Empty CFD model	7.21	0.99	6.32
Atom. Therm. model	2.15	0.99	1.74
Atom. CFD model	14.91	0.69	10.43

## Data Availability

Data is contained within the article and supplementary material.

## Supplementary Materials

The following are available online at https://www.mdpi.com/article/10.3390/pharmaceutics14020296/s1, Equations (S1) and (S2) proof: equation6.pdf.

## Abbreviations

The following abbreviations have been used in this manuscript:

CFD	Computational fluid dynamics
ML	Machine learning
$T_{\mathrm{in}}$	Inlet temperature of the drying gas (K)
$T_{\mathrm{out}}$	Outlet temperature of the drying gas (K)
$T_{\mathrm{ext}}$	Environmental temperature (K)
$T_{\mathrm{avg}}$	Average temperature of inlet and outlet (K)
$T_{\mathrm{ext}}^{\mathrm{wall}}$	Temperature of the spray dryer’s outer surface (K)
$T_{g}$	Glass-transition temperature (K)
$RH_{\mathrm{ext}}$	Environmental relative humidity
$G_{\mathrm{in}}$	Drying gas flow rate (kg/h)
$G_{\mathrm{in}}^{m}$	Drying gas speed (m/s)
FR	Feed flow rate (kg/h)
$c_{\mathrm{feed}}$	Concentration of the feed
$Q_{\mathrm{in}}^{}$	Heat entering spray drying (kJ/h)
$Q_{\mathrm{out}}^{}$	Heat coming out of the equipment (kJ/h)
$Q_{\mathrm{feed}}$	Heat required to dry the feed (kJ/h)
$Q_{\mathrm{loss}}$	Heat lost to the external environment (kJ/h)
$Q_{\mathrm{loss}}^{R}$	Heat lost by convection and conduction (kJ/h)
$Q_{\mathrm{loss}}^{\mathrm{rad}}$	Heat lost by radiation (kJ/h)
$cp_{\mathrm{gas}}$	Thermal conductivity of the air (kJ/kg·K)
${\mathrm{emissivity}}_{\mathrm{glass}}$	Heat emissivity coefficient of the glass
${\mathrm{Area}}_{\mathrm{ext}}$	External surface of the semplified spray dryer (m${}^{2}$)
