# Supplementary material for: Thermodynamic Balance vs. Computational Fluid Dynamics Approach for the Outlet Temperature Estimation of a Benchtop Spray Dryer
Source: Pharmaceutics. 2022 Jan 27;14(2):296. doi: 10.3390/pharmaceutics14020296 (PMC8877328; doi:10.3390/pharmaceutics14020296)
Supplement: Supplementary file 1 [file pharmaceutics-14-00296-s001.zip › pharmaceutics-1515125-supplementary.pdf]

Supplementary Materials: Thermodynamic Balance vs. Computational Fluid Dynamics Approach for the Outlet Temperature Estimation of a Benchtop Spray Dryer

Andrea Milanesi, Francesco Rizzuto, Maurizio Rinaldi, Andrea Foglio Bonda, Lorena Segale and Lorella Giovannelli *

Proof. To obtain Equation (6) it is possible to start from Q_{feed} (S1) and Q_{loss} (S2):

$$Q_{feed}(T_{in}, T_{ext}, RH_{ext}, FR, c_{feed}) = (FR \cdot (1 - c_{feed})) \cdot (H_{evap}(T_{ext}) + cp_{feed}(T_{in}) \cdot (T_{wetbulb}(T_{ext}, RH_{ext}) - T_{ext})) \quad (S1)$$

(see Lisboa et al. [1])

Q_{loss} as reported by Çengel [2] can be decomposed as:

$$Q_{loss}(T_{in}, T_{ext}, T_{ext}^{wall}, T_{out}, G_{in}^{m}) = Q_{loss}^{R}(T_{in}, T_{ext}, T_{out}, G_{in}^{m}) + Q_{loss}^{rad}(T_{ext}^{wall}, T_{ext}) \quad (S2)$$

where:

$$Q_{loss}^{R}(T_{in}, T_{ext}, T_{out}, G_{in}^{m}) = \frac{T_{in} - T_{ext}}{R(T_{in}, T_{out}, G_{in}^{m})} \cdot \text{Tower}_{length} \quad (S3)$$

$$Q_{loss}^{rad}(T_{ext}^{wall}, T_{ext}) = K \cdot ({T_{ext}^{wall}}^{4} - T_{ext}^{4}) \quad (S4)$$

$$K = \text{Area}_{ext} \cdot \sigma \cdot \text{emissivity}_{glass} \quad (S5)$$

It is possible to obtain Equation (6) in 4 steps:

1. Q_{loss} can be substituted in Equation (5) with Equation (S2) getting:

$$T_{out} = T_{in} - \frac{Q_{feed} + Q_{loss}^{R} + Q_{loss}^{rad}}{cp_{gas} \cdot G_{in}} \quad (S6)$$

2. Q_{loss}^{rad} can be deduced from Equation (S6)

$$Q_{loss}^{rad} = (T_{in} - T_{out}) \cdot cp_{gas} \cdot G_{in} - Q_{feed} - Q_{loss}^{R} \quad (S7)$$

3. Q_{loss}^{rad} equation (S4) can be inverted to find T_{ext}^{wall}

$$T_{ext}^{wall} = \left(\frac{Q_{loss}^{rad}}{K} + T_{ext}^{4}\right)^{1/4} \quad (S8)$$

4. And finally it is possible to substitute Q_{loss}^{rad} of (S7) in (S8) getting:

$$T_{ext}^{wall} = \left(\frac{(T_{in} - T_{out}) \cdot cp_{gas} \cdot G_{in} - Q_{feed} - Q_{loss}^{R}}{K} + T_{ext}^{4}\right)^{1/4} \quad (S9)$$

On the T_{ext}^{wall} values can be apply the ML.

□

References

1. Lisboa, H.M.; Duarte, M.E.; Cavalcanti-Mata, M.E. Modeling of food drying processes in industrial spray dryers. *Food Bioprod. Process.* **2018**, *107*, 49–60.
2. Çengel, Y.A. Introduction to Thermodynamics and Heat Transfer; Çengel series in engineering for the thermal-fluid sciences. McGraw-Hill: New York, NY, USA, 2009.
